# Supplementary material for: Nucleation feedback can drive establishment and maintenance of biased microtubule polarity in neurites
Source: ArXiv. 2025 Aug 28:arXiv:2506.12209v2. Preprint. [Version 2] (PMC12407633)
Supplement: Supplement 1 [file NIHPP2506.12209v2-supplement-1.pdf]

A Supplemental figures

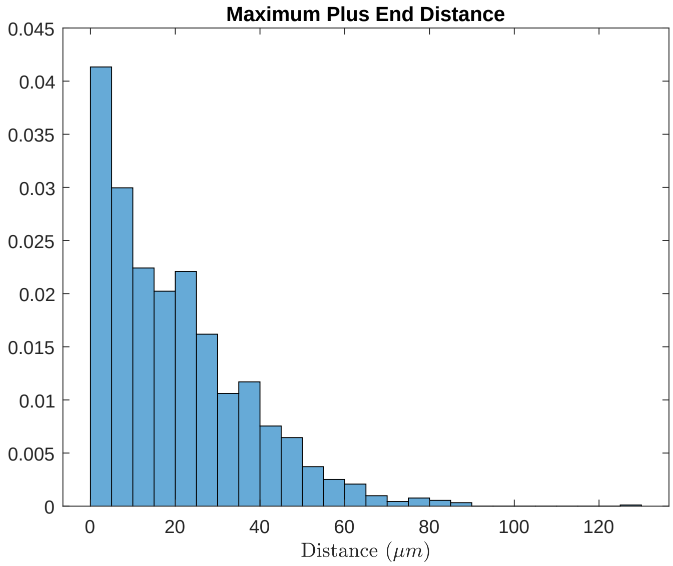

Figure 9: Distribution of the maximum distance reached by MT plus ends for 1829 simulations of full MT lifetimes, from nucleation to complete catastrophe, in the MT polymerization model. This base model does not include spatial constraints or nucleation control mechanisms. Each MT is initialized with a seed length of 0.1 μm. The fraction of MTs that grew at least 2 μm is 0.9076.

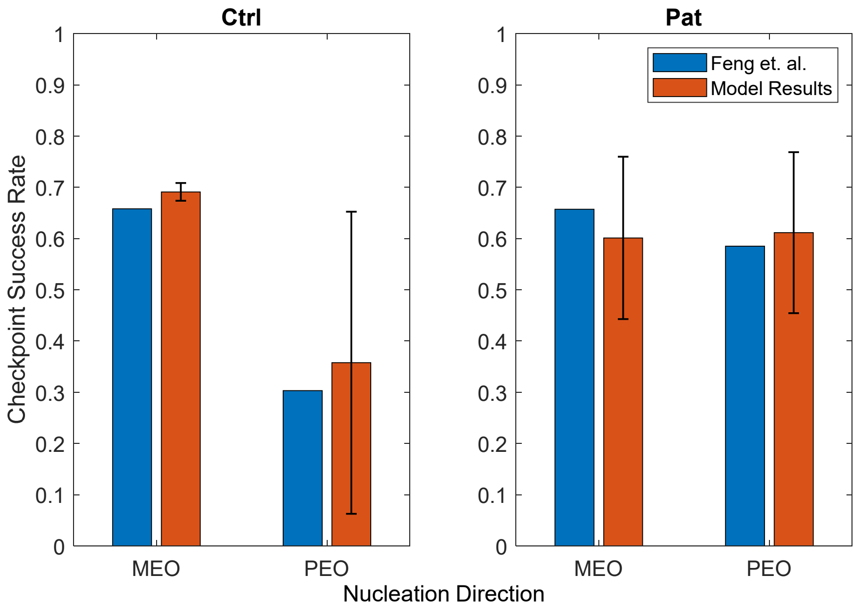

Figure 10: Model checkpoint success rates comparing results from [9] (blue) to average model results (red) with standard deviation error bars. Success rates were computed from 10 independent 30-hour simulations of the long model domain initialized with 90% MEO and 50% MEO MTs for the control and Patronin environments, respectively. MTs were counted as successfully passing a checkpoint if they pass the checkpoint autofail, they satisfy the matching pre-existing content condition, and they grew to be at least 2 μm long. Otherwise, they were counted as failures.

B Supplemental video captions

B.1 Supplemental video 1

Two example simulations of our single nucleation location domain with MEO MTs in pink and PEO MTs in blue. These result in fully-biased MEO (left) and PEO (right) polarity. For both simulations, both nucleation feedback and checkpoint mechanisms were implemented. MTs are visualized from oldest to newest (based on time of nucleation) from left to right.

B.2 Supplemental video 2

Three example simulations of our long domain with 6 nucleation locations with MEO MTs in pink and PEO MTs in blue. These demonstrate spatial polarity phase separation, achieve different fully-biased polarity states, and depart fully-biased polarity states. For all simulations, both nucleation feedback and checkpoint mechanisms were implemented. MTs are visualized from oldest to newest (based on time of nucleation) from left to right.
